# Echinoids of the Kerguelen Plateau – occurrence data and environmental setting for past, present, and future species distribution modelling

**DOI:** 10.3897/zookeys.630.9856

**Published:** 2016-11-09

**Authors:** Charlène Guillaumot, Alexis Martin, Salomé Fabri-Ruiz, Marc Eléaume, Thomas Saucède

**Affiliations:** 1 UMR 6282 Biogéosciences, Univ. Bourgogne Franche-Comté, CNRS, 6 bd Gabriel F-21000 Dijon, France; 2 Muséum national d’Histoire naturelle, Département Milieux et Peuplements Aquatiques, UMR BOREA 7208, 57 rue Cuvier, F-75231 Paris Cedex 05, France; 3 Muséum national d’Histoire naturelle, Département Systématique et Évolution, UMR ISYEB 7205, 57 rue Cuvier, F-75231 Paris Cedex 05, France

**Keywords:** Echinoidea, environmental descriptors, future projections, historical overview, Kerguelen Plateau, Southern Ocean

## Abstract

The present dataset provides a case study for species distribution modelling (SDM) and for model testing in a poorly documented marine region.

species distribution modelling

The dataset includes spatially-explicit data for echinoid (Echinodermata: Echinoidea) distribution. Echinoids were collected during oceanographic campaigns led around the Kerguelen Plateau (+63°/+81°E; -46°/-56°S) since 1872. In addition to the identification of collection specimens from historical cruises, original data from the recent campaigns POKER II (2010) and PROTEKER 2 to 4 (2013-2015) are also provided. In total, five families, ten genera, and 12 echinoid species are recorded in the region of the Kerguelen Plateau.

The dataset is complemented with environmental descriptors available and relevant for echinoid ecology and SDM. The environmental data was compiled from different sources and was modified to suit the geographic extent of the Kerguelen Plateau, using scripts developed with the R language (R Core Team 2015). Spatial resolution was set at a common 0.1° pixel resolution. Mean seafloor and sea surface temperatures, salinity and their amplitudes, all derived from the World Ocean Database (Boyer et al. 2013) are made available for the six following decades: 1955–1964, 1965–1974, 1975–1984, 1985–1994, 1995–2004, 2005–2012.

Future projections are provided for several parameters: they were modified from the Bio-ORACLE database (Tyberghein et al. 2012). They are based on three IPCC scenarii (B1, AIB, A2) for years 2100 and 2200 (IPCC, 4^th^ report).

## Project description

**Project title**: Temporal, spatial, and sampling heterogeneities in species distribution modelling. A case study for the data-poor area of the Kerguelen Plateau.

**Personnel**: Charlène Guillaumot, Alexis Martin, Salomé Fabri-Ruiz, Marc Eléaume, Thomas Saucède

**Funding**: This study is part of a project funded by CNRS laboratory UMR6282 Biogeosciences and by the vERSO program (Ecosystem Responses to global change: a multiscale approach in the Southern Ocean). This is contribution no.14 to the vERSO project (www.versoproject.be), funded by the Belgian Science Policy Office (BELSPO, contract n°BR/132/A1/vERSO). This is a contribution to the POKER program and the IPEV (Institut polaire français Paul-Emile Victor) program 1044 PROTEKER.

### Study extent description

The study area of this dataset includes the Kerguelen Plateau, located at the boundary between the Indian and Southern Oceans, in the flow of the Antarctic Circumpolar Current (Park and Vivier 2011). The plateau is the second largest oceanic igneous province on Earth. It is positioned between 46°S and 62°S latitude, between 63°E and 81°E longitude, and it extends over 500 km from East to West and 2,100 km from North to South for a total surface area of 2.10^6^ km^2^ (Cottin et al. 2011).

The Kerguelen Plateau is subdivided into the Kerguelen Islands shelf in the north and the Heard and McDonalds Islands shelf in the south. The two shelves are separated by a controlling oceanographic barrier: the Polar Front, which position has recurrently been discussed (Park et al. 2014). Topography and currents also strongly control other environmental parameters (temperature, salinity, chlorophyll a concentration) in the vicinity of the Plateau (Graham et al. 2012, Chacko et al. 2014).

The Kerguelen Plateau hosts important economic activities, namely through fishing, generating potential issues for the conservation of marine biodiversity. Exploitation of the marine living resources of the Kerguelen Plateau has been sustainably managed by CCAMLR (Commission for the Conservation of Antarctic Marine Living Resources) and by the TAAF (French Southern and Antarctic Lands) in the French EEZ (Exclusive Economic Zone) with scientific support from the Muséum national d’Histoire naturelle of Paris since 1978 (Duhamel and Williams 2011). In the Australian EEZ, in the south, a similar management system was established in 1979 and was followed by the designation in 2002 of the Heard Island and McDonald Islands PageBreak(HIMI) Marine Protected Area: one of the world’s largest MPA with an area of 65,000 km^2^ (Welsford et al. 2011).

The Kerguelen Plateau represents a vast marine area challenged by strong anthropogenic and natural pressures. Relatively few scientific programs have studied marine biodiversity of the Kerguelen Plateau, leaving it poorly documented. In this context, environmental descriptors could prove to be useful proxies to infer species distribution when occurrence data are missing (Hemery et al. 2011).

In addition to the study of collection specimens sampled during historical cruises and identified at species level, the present work also provides original data collected during the recent oceanographic campaign POKER II (2010) and during three field summer campaigns of the IPEV program 1044 PROTEKER (2013-2015) led in nearshore areas of the Kerguelen Islands. The spatial extent of the dataset was based on the bathymetric range of echinoids for species distribution modelling to be performed with limited extrapolations.

### Design description

Our project aimed at improving the robustness of existing modelling approaches in the case of areas for which only poor and heterogeneous biodiversity data are available, a situation prevailing in the region of the Kerguelen Plateau, and generally in the Southern Ocean (Gutt et al. 2012).

Data compilation from various sources implies temporal heterogeneities that may constitute a critical point when building species distribution models (Aguiar et al. 2015). Spatial and sampling heterogeneities are also likely to introduce biases due to differences in sampling strategies and the gears used during the various cruises. Our objectives were (1) to assess the influence of temporal, spatial, and sampling heterogeneities on species distribution modelling using datasets of echinoid occurrences on the Kerguelen Plateau, (2) to model echinoid distribution on the Kerguelen Plateau for different time periods, and (3) to evaluate potential shifts in species distribution with regards to future projections based on IPCC scenarii (Jueterbock et al. 2013).

### Data description

Occurrence data were compiled from many oceanographic campaigns led over a long time-period starting with the Challenger Expedition in 1872 and ending with the recent PROTEKER campaigns that took place between 2013 and 2015 (Table 1). The dataset was modified after Pierrat et al. (2012) and Saucède et al. (2015a). Specimens from recent cruises (POKER II and PROTEKER) were identified at species level and added to the dataset.

**Table 1.** Field campaigns during which echinoids of the dataset were collected. MNHN: Muséum national d’Histoire naturelle.

Campaigns	Year	Occurrence Nb	Research vessels	References	Collections
Challenger Expedition	1872	9	Challenger	A. Agassiz 1879, 1881	National History Museum, London
Gazelle Expedition	1874–76	1	Gazelle	Studer 1876	Museum für Naturkunde, Berlin
Deutsche Tiefsee Expedition	1898–99	3	Valdivia	Döderlein 1906	Museum für Naturkunde, Berlin
Deutsche Südpolar Expedition	1901–03	2	Gauss	Mortensen 1909	Museum für Naturkunde, Berlin
BANZAR Expedition	1929	9	Discovery	Mortensen 1950	National Museum of Australia, Acton
Kerguelen 1962–63	1962–63	8	-	Grua 1963	MNHN, Paris
Eltanin Expedition	1962–72	1	Eltanin	Fell 1976	Smithsonian Institution, Washington
Ker72	1972	5	Japonaise	Guille 1977	MNHN, Paris
MD03	1974	32	Marion Dufresne	De Ridder et al. 1992	MNHN, Paris
MD04	1975	130	Marion Dufresne	De Ridder et al. 1992	MNHN, Paris
SIBEX MD42	1985	13	Marion Dufresne	Pierrat et al. 2012	MNHN, Paris
1985 ANARE Expedition	1985	5	Nella Dan	Burton and Williams 1985	National Museum of Australia, Acton
Benthos/mac	1991	22	Curieuse	Poulin and Féral 1995	Banyuls oceanological observatory
1992 ANARE Expedition	1992	18	Aurora Australis	Green 1993	National Museum of Australia, Acton
Cruise SC26	2003	2	Southern Champion	Pierrat et al. 2012	Australian Antarctic Division, Kingston
POKER II	2010	111	Austral	this study	MNHN, Paris
PROTEKER 2	2013	52	Curieuse	Féral et al. 2013	MNHN, Paris
PROTEKER 3	2014	7	Curieuse	Féral et al. 2014	MNHN, Paris
PROTEKER 4	2015	5	Commerson	Saucède et al. 2015b	MNHN, Paris

**Figure 1.**
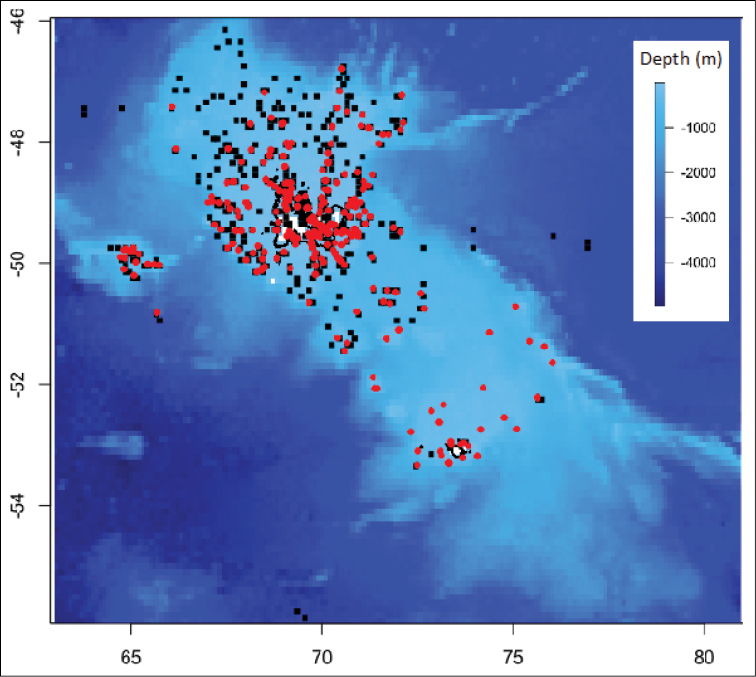
Sampling effort. Red dots depict echinoid occurrences. Black squares correspond to visited sites at which no echinoid was sampled.

Occurrences are presence-only data for which different sampling tools, protocols, and strategies were used. Moreover, the study area was unevenly investigated, sampling effort being stronger in the northern than in the southern part of the Plateau (Figure 1). Accordingly, campaigns and sampling dates are mentioned in the dataset to take into account spatial and time heterogeneities.

The environmental descriptors provided in the dataset were compiled from different sources (Table 2 – see Annex). They were selected according to their relevance to echinoid ecology.

Environmental data were formatted with R3.3.0 software (R Core Team 2015) to fit the sampling area of where echinoids occur on the Kerguelen Plateau (+63°/+81°E; -46°/-56°S). They were set up to a 0.1° grid-cell spatial resolution with origin fixed at 0 (top left corner). Seafloor temperature, salinity, oxygen and nutrient concentration data were generated by using the provided data of the World Ocean Database (Boyer et al. 2013) and depth data.

**Table 2.** Environmental variables provided in the present data paper. Salinity data are reported in the Practical Salinity Scale (PSS) format.

Environmental layer name	Spatial cover	Unit	Source	URL
seafloor_salinity_amplitude_1955_1964	46_56°S/63_81°E	PSS	This study. Derived from World Ocean Database (Boyer et al. 2013) surface salinity layers	https://www.nodc.noaa.gov/OC5/woa13/woa13data.html
seafloor_salinity_amplitude_1965_1974	46_56°S/63_81°E	PSS		
seafloor_salinity_amplitude_1975_1984	46_56°S/63_81°E	PSS		
seafloor_salinity_amplitude_1985_1994	46_56°S/63_81°E	PSS		
seafloor_salinity_amplitude_1995_2004	46_56°S/63_81°E	PSS		
seafloor_salinity_amplitude_2005_2012	46_56°S/63_81°E	PSS		
seafloor_salinity_amplitude_1955_2012	46_56°S/63_81°E	PSS		
seasurface_salinity_amplitude_1955_1964	46_56°S/63_81°E	PSS	World Ocean Database (Boyer et al. 2013)	
seasurface_salinity_amplitude_1965_1974	46_56°S/63_81°E	PSS		
seasurface_salinity_amplitude_1975_1984	46_56°S/63_81°E	PSS		
seasurface_salinity_amplitude_1985_1994	46_56°S/63_81°E	PSS		
seasurface_salinity_amplitude_1995_2004	46_56°S/63_81°E	PSS		
seasurface_salinity_amplitude_2005_2012	46_56°S/63_81°E	PSS		
seasurface_salinity_amplitude_1955_2012	46_56°S/63_81°E	PSS		
seasurface_temperature_amplitude_2100_A1B	46_56°S/63_81°E	°Celsius degrees	BIO-ORACLE (Tyberghein et al. 2012)	http://www.oracle.ugent.be/
seasurface_temperature_amplitude_2100_A2	46_56°S/63_81°E	°Celsius degrees		
seasurface_temperature_amplitude_2100_B1	46_56°S/63_81°E	°Celsius degrees		
seasurface_temperature_amplitude_2200_A1B	46_56°S/63_81°E	°Celsius degrees		
seasurface_temperature_amplitude_2200_B1	46_56°S/63_81°E	°Celsius degrees		
seafloor_temperature_amplitude_1955_1964	46_56°S/63_81°E	°Celsius degrees	This study. Derived from World Ocean Database (Boyer et al. 2013) sea surface temperature layers	https://www.nodc.noaa.gov/OC5/woa13/woa13data.html
seafloor_temperature_amplitude_1965_1974	46_56°S/63_81°E	°Celsius degrees		
seafloor_temperature_amplitude_1975_1984	46_56°S/63_81°E	°Celsius degrees		
seafloor_temperature_amplitude_1985_1994	46_56°S/63_81°E	°Celsius degrees		
seafloor_temperature_amplitude_1995_2004	46_56°S/63_81°E	°Celsius degrees		
seafloor_temperature_amplitude_2005_2012	46_56°S/63_81°E	°Celsius degrees		
seafloor_temperature_amplitude_1955_2012	46_56°S/63_81°E	°Celsius degrees		
seasurface_temperature_amplitude_1955_1964	46_56°S/63_81°E	°Celsius degrees	World Ocean Database (Boyer et al. 2013)	
seasurface_temperature_amplitude_1965_1974	46_56°S/63_81°E	°Celsius degrees		
seasurface_temperature_amplitude_1975_1984	46_56°S/63_81°E	°Celsius degrees		
seasurface_temperature_amplitude_1985_1994	46_56°S/63_81°E	°Celsius degrees	World Ocean Database (Boyer et al. 2013)	https://www.nodc.noaa.gov/OC5/woa13/woa13data.html
seasurface_temperature_amplitude_1995_2004	46_56°S/63_81°E	°Celsius degrees		
seasurface_temperature_amplitude_2005_2012	46_56°S/63_81°E	°Celsius degrees		
seasurface_temperature_amplitude_1955_2012	46_56°S/63_81°E	°Celsius degrees		
chlorophyla_summer_mean_2002_2009	46_56°S/63_81°E	mg/m^3^	MODIS AQUA (NASA) 2010	http://gdata1.sci.gsfc.nasa.gov/daac-bin/G3/gui.cgi?instance_id=ocean_8day
geomorphology	46_56°S/63_81°E	categorial	ATLAS ETOPO2 2014 (Douglass et al. 2014)	
depth	46_56°S/63_81°E	meter	This study. Derived from Smith and Sandwell 1997	http://topex.ucsd.edu/WWW_html/mar_topo.html
seafloor_nitrate_mean_1955_2012	46_56°S/63_81°E	µmol/L	This study. Derived from World Ocean Database (Boyer et al. 2013) sea surface nitrate concentration layers	https://www.nodc.noaa.gov/OC5/woa13/woa13data.html
seasurface_nitrate_mean_1955_2012	46_56°S/63_81°E	µmol/L	World Ocean Circulation Experiment 2013	
seafloor_oxygen_mean_1955_2012	46_56°S/63_81°E	mL/L	This study. Derived from World Ocean Database (Boyer et al. 2013) sea surface oxygen concentration layers	
seasurface_oxygen_mean_1955_2012	46_56°S/63_81°E	mL/L	World Ocean Circulation Experiment 2013	
seafloor_phosphate_mean_1955_2012	46_56°S/63_81°E	µmol/L	This study. Derived from World Ocean Database (Boyer et al. 2013) sea surface phosphate concentration layers	
seasurface_phosphate_mean_1955_2012	46_56°S/63_81°E	µmol/L	World Ocean Circulation Experiment 2013	
roughness	46_56°S/63_81°E	meter	This study. Derived from bathymetric layer	
seafloor_salinity_mean_1955_1964	46_56°S/63_81°E	PSS	This study. Derived from World Ocean Database (Boyer et al. 2013) sea surface salinity layers	https://www.nodc.noaa.gov/OC5/woa13/woa13data.html
seafloor_salinity_mean_1965_1974	46_56°S/63_81°E	PSS		
seafloor_salinity_mean_1975_1984	46_56°S/63_81°E	PSS		
seafloor_salinity_mean_1985_1994	46_56°S/63_81°E	PSS		
seafloor_salinity_mean_1995_2004	46_56°S/63_81°E	PSS		
seafloor_salinity_mean_2005_2012	46_56°S/63_81°E	PSS		
seafloor_salinity_mean_1955_2012	46_56°S/63_81°E	PSS		
seasurface_salinity_mean_1955_1964	46_56°S/63_81°E	PSS	World Ocean Database (Boyer et al. 2013)	
seasurface_salinity_mean_1965_1974	46_56°S/63_81°E	PSS		
seasurface_salinity_mean_1975_1984	46_56°S/63_81°E	PSS		
seasurface_salinity_mean_1985_1994	46_56°S/63_81°E	PSS		
seasurface_salinity_mean_1995_2004	46_56°S/63_81°E	PSS		
seasurface_salinity_mean_2005_2012	46_56°S/63_81°E	PSS	World Ocean Database (Boyer et al. 2013)	https://www.nodc.noaa.gov/OC5/woa13/woa13data.html
seasurface_salinity_mean_1955_2012	46_56°S/63_81°E	PSS		
seasurface_salinity_mean_2100_A1B	46_56°S/63_81°E	PSS	BIO-ORACLE (Tyberghein et al. 2012)	http://www.oracle.ugent.be/
seasurface_salinity_mean_2100_A2	46_56°S/63_81°E	PSS		
seasurface_salinity_mean_2100_B1	46_56°S/63_81°E	PSS		
seasurface_salinity_mean_2200_A1B	46_56°S/63_81°E	PSS		
seasurface_salinity_mean_2200_B1	46_56°S/63_81°E	PSS		
sediments	46_56°S/63_81°E	categorial	McCoy (1991), updated by Griffiths 2014 (unpublished)	
seafloor_silicate_mean_1955_2012	46_56°S/63_81°E	µmol/L	This study. Derived from World Ocean Database (Boyer et al. 2013) sea surface silicate concentration layers	https://www.nodc.noaa.gov/OC5/woa13/woa13data.html
seasurface_silicate_mean_1955_2012	46_56°S/63_81°E	µmol/L	World Ocean Circulation Experiment 2013	
slope	46_56°S/63_81°E	unitless	Smith and Sandwell 1997	
seafloor_temperature_mean_1955_1964	46_56°S/63_81°E	°Celsius degrees	This study. Derived from World Ocean Database (Boyer et al. 2013) sea surface temperature layers	https://www.nodc.noaa.gov/OC5/woa13/woa13data.html
seafloor_temperature_mean_1965_1974	46_56°S/63_81°E	°Celsius degrees		
seafloor_temperature_mean_1975_1984	46_56°S/63_81°E	°Celsius degrees		
seafloor_temperature_mean_1985_1994	46_56°S/63_81°E	°Celsius degrees		
seafloor_temperature_mean_1995_2004	46_56°S/63_81°E	°Celsius degrees		
seafloor_temperature_mean_2005_2012	46_56°S/63_81°E	°Celsius degrees		
seafloor_temperature_mean_1955_2012	46_56°S/63_81°E	°Celsius degrees		
seasurface_temperature_mean_1955_1964	46_56°S/63_81°E	°Celsius degrees	World Ocean Database (Boyer et al. 2013)	
seasurface_temperature_mean_1965_1974	46_56°S/63_81°E	°Celsius degrees		
seasurface_temperature_mean_1975_1984	46_56°S/63_81°E	°Celsius degrees		
seasurface_temperature_mean_1985_1994	46_56°S/63_81°E	°Celsius degrees		
seasurface_temperature_mean_1995_2004	46_56°S/63_81°E	°Celsius degrees		
seasurface_temperature_mean_2005_2012	46_56°S/63_81°E	°Celsius degrees		
seasurface_temperature_mean_1955_2012	46_56°S/63_81°E	°Celsius degrees		
seasurface_temperature_mean_2100_A1B	46_56°S/63_81°E	°Celsius degrees	BIO-ORACLE (Tyberghein et al. 2012)	http://www.oracle.ugent.be/
seasurface_temperature_mean_2100_A2	46_56°S/63_81°E	°Celsius degrees		
seasurface_temperature_mean_2100_B1	46_56°S/63_81°E	°Celsius degrees		
seasurface_temperature_mean_2200_A1B	46_56°S/63_81°E	°Celsius degrees		
seasurface_temperature_mean_2200_B1	46_56°S/63_81°E	°Celsius degrees		

In marine nearshore areas, grid-cells with positive depth values above sea level were corrected for accuracy using ArcGis Raster Editor Tool (ESRI 2011) based on geographic charts (IGN: National Geographic Institute, EAN: 3282110102707, scale 1/200 000) and raw depth values measured in the field (Féral et al. 2013, 2014, Saucède et al. 2015b).

Roughness data were computed using the “terrain” function of the raster package R3.3.0 (Hijmans and van Etten 2012).

The time coverage of the environmental data extends from 1955 to 2012. Mean annual surface and seafloor temperatures, salinity and their respective amplitudes (i.e., amplitude between mean summer (January to March) and mean winter (July to September) surface and seafloor temperatures and salinities) are available for the following six decades: 1955 to 1964, 1965 to 1974, 1975 to 1984, 1985 to 1994, 1995 to 2004, and 2005 to 2014.

Future projections of sea surface temperature, salinity, and amplitude were downloaded from the Bio-ORACLE database (Tyberghein et al. 2012). Projections are based on the IPCC A2, A1B, and B1 scenarii published in the 4^th^ IPCC report (2007). The modelled data correspond to the extrapolated means for two decades: 2087-2096 (here referred to as 2100) and 2187-2196 (here referred to as 2200) (Jueterbock et al. 2013).

All the environmental descriptors and metadata sources are detailed in the data catalog (Table 2) and data are provided in an ascii raster format. N/A was set as the no data reference for missing data.

### Quality control description

Specimens sampled during POKER II and PROTEKER 2, 3 and 4 campaigns were all identified by T. Saucède at the species level. Identifications and taxonomic accuracies are based on Anderson (2009), Anderson (2012), David et al. (2005), Kroh and Smith (2010), Pierrat et al. (2012), and Saucède et al. (2015a).

The final compiled dataset was checked for consistency using the WoRMS database (WoRMS Editorial Board 2016) in order to match our data with the most up-to-date taxonomy. The dataset was checked for duplicates and errors due to overlapping origins, georeferencing mistakes, and species synonymy or mis-spelling. Only occurrence data identified at the species level were included.

Environmental data relies on different sources as reported in Table 2. The range of data was studied to check for variables consistencies. Data were not interpolated to limit interpolation biases and missing data were reported as N/A values.

## Taxonomic coverage

### General taxonomic coverage description:

The present dataset focuses on all species of the class Echinoidea (Echinodermata) occurring on the Kerguelen Plateau.

Echinoids are common species of benthic communities in the Southern Ocean and on the Kerguelen Plateau (David et al. 2005). They are diversified and well-studied. Historical data are available since 1872, starting with the Challenger Expedition, and are completed with recent occurrences collected nearshore areas of the Kerguelen Islands during the PROTEKER campaigns (2013-2015).

Echinoid studies take part in conservation issues. *Ctenocidaris nutrix* is considered a Vulnerable Marine Ecosystems (VME) indicator species by CCAMLR (Commission for the Conservation of Antarctic Marine Living Resources) and is widely distributed on the Kerguelen Plateau.

**Figure 2.**
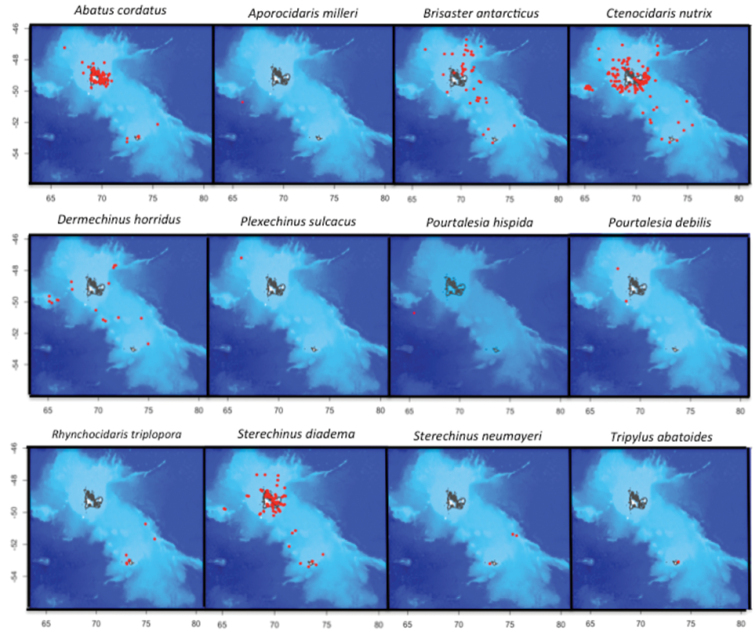
Distribution of the 12 echinoid species based on the specimens collected since 1872 on the Kerguelen Plateau.

On the Kerguelen Plateau, the Class Echinoidea includes five families, ten genera, and 12 species. Species distribution is shown in Figure 2.

**Phylum**: Echinodermata

**Class**: Echinoidea

**Order**: Camarodonta, Cidaroida, Holasteroida, Spatangoida

**Family**: Ctenocidarinae, Echinidae, Plexechinidae, Pourtalesiidae, Schizasteridae

**Genus**: *Abatus*, *Aporocidaris*, *Brisaster*, *Ctenocidaris*, *Dermechinus*, *Plexechinus*, *Pourtalesia*, *Rhynchocidaris*, *Sterechinus*, *Tripylus*

**Species**: *Abatus cordatus*, *Aporocidaris milleri*, *Brisaster antarcticus*, *Ctenocidaris nutrix*, *Dermechinus horridus*, *Plexechinus sulcatus*, *Pourtalesia hispida*, *Pourtalesia debilis*, *Rhynchocidaris triplopora*, *Sterechinus diadema*, *Sterechinus neumayeri*, *Tripylus abatoides*

### Spatial coverage

General spatial coverage: the Kerguelen Plateau, Southern Ocean

Coordinates: -46°S and -56°S; +63°E and +81°E

### Temporal coverage

Temporal coverage: 1872–2015

## Datasets

### Dataset occurrence description

Echinoid occurrences available on the Kerguelen Plateau. Data from 1872 to 2015 collected with different sampling strategies and objectives, during different campaigns.

**Object name**: Echinoids_Kerguelen_Plateau_1872_2015

**Character encoding**: x-MacRoman

**Format name**: Darwin Core Archive Format

**Format version**: 3.0

**Distribution**: http://ipt.biodiversity.aq/resource.do?r=echinoids_kerguelen_plateau_18- 72_2015

**Publication date of data**: 12/07/2016

**Language**: English

**Metadata language**: English

**Date of metadata creation**: 12/07/2016

**Hierarchy level**: Dataset

### Dataset of actual environmental parameters description

Environmental variables in the region of the Kerguelen Plateau compiled from different sources and provided in the ascii raster format (Guillaumot et al. 2016). Mean surface and seafloor temperature, salinity and their respective amplitude data are available on the time coverage 1955-2012 and over six decades: 1955 to 1964, 1965 to 1974, 1975 to 1984, 1985 to 1994 and 1995 to 2004, and 2005 to 2012.

Future projections are provided for several parameters: they were modified after the Bio-ORACLE database (Tyberghein et al. 2012). They are based on three IPCC scenarii (B1, AIB, A2) for years 2100 and 2200 (IPCC, 4^th^ report).

**Object name**: Environmental_Kerguelen_Plateau_1955_2012

**Format name**: Raster

**Format version**: 1.0

**Distribution**: https://data.aad.gov.au/metadata/records/Environmental_Kerguelen_Plateau_1955_2012

**doi**: 10.4225/15/578ED5A08050F

**Publication date of data**: 16/07/2016

**Language**: English

**Metadata language**: English

**Date of metadata creation**: 16/07/2016

**Hierarchy level**: Dataset
